# Daptomycin versus Glycopeptides for the Treatment of *Enterococcus faecium* Bacteraemia: A Cohort Study

**DOI:** 10.3390/antibiotics10060716

**Published:** 2021-06-14

**Authors:** Daniel Echeverría-Esnal, Luisa Sorli, Nuria Prim, Clara Martin-Ontiyuelo, Juan Pablo Horcajada, Santiago Grau

**Affiliations:** 1 Pharmacy Department, Hospital del Mar, Passeig Maritim 25–29, 08003 Barcelona, Spain; dechevarria@psmar.cat; 2 Infectious Pathology and Antimicrobials Research Group (IPAR), Institut Hospital del Mar d’Investigacions Mèdiques (IMIM), Dr. Aiguader 88, 08003 Barcelona, Spain; lsorli@psmar.cat (L.S.); jhorcajada@psmar.cat (J.P.H.); 3 Infectious Diseases Department, Hospital del Mar, Passeig Maritim 25–29, 08003 Barcelona, Spain; 4 Microbiology Department, Laboratori de Referència de Catalunya, Carrer de la Selva 10, 08820 Barcelona, Spain; nprim@lrc.es; 5 Pneumology Department, Hospital del Mar, Passeig Marítim 25, 08003 Barcelona, Spain; CMartinOntiyuelo@parcdesalutmar.cat; 6 Department of Medicine, CEXS-Universitat Pompeu Fabra, 08003 Barcelona, Spain; 7 Department of Pharmacology, Universitat Autònoma de Barcelona, 08193 Barcelona, Spain

**Keywords:** *Enterococcus faecium*, bloodstream infection, bacteraemia, daptomycin, glycopeptides, vancomycin

## Abstract

Background: Ampicillin resistant and glycopeptide susceptible *Enterococcus faecium* bloodstream infection (GSEF-BSI) incidence has risen. However, the treatment of choice remains unknown. Daptomycin use for the treatment of enterococcal infections has increased, despite effectiveness and safety concerns. The objective was to compare the effectiveness and safety of daptomycin and glycopeptides in the treatment of GSEF-BSI. Methods: This was a single-centre, retrospective observational cohort study performed at Hospital del Mar (Barcelona, Spain), from January 2006–May 2018. The primary outcome was clinical cure at the end of the therapy, and secondary outcomes included 14-day, 30-day, in-hospital mortality, and length of stay. Results: From a total of 192 patients with GSEF-BSI, 54 (28.1%) were treated with glycopeptides and 17 (8.9%) with daptomycin. Patients treated with daptomycin presented a lower clinical cure than patients treated with glycopeptides (58.8% vs. 83.3%, RR 0.416 (95% CI 0.189–0.915)). After controlling for confounding variables by means of multivariate analysis the significative difference was confirmed (aOR 4.313, 95% CI, 1.053–17.660). The need for treatment discontinuation due to adverse events was similar. Conclusions: Patients with GSEF-BSI treated with glycopeptides showed a higher clinical cure than those treated with daptomycin.

## 1. Introduction

Enterococci are ubiquitous Gram-positive bacteria that have been isolated from a wide variety of places, including the gut microbiota [1]. Although these microorganisms are considered commensal organisms of the gastrointestinal tract, they can cause serious infections such as bloodstream infections (BSI) or endocarditis, and have emerged as important nosocomial pathogens [1,2]. They are the second most common cause of healthcare associated infections in Europe and the United States of America (USA) [1].

Currently, there are two main species among enterococci that can cause infections: *Enterococcus faecalis* and *Enterococcus faecium* [1]. Although *E. faecalis* is more virulent and is the main enterococci involved in human infections, the incidence of *E. faecium* infections is rising [1]. Concerning enterococcal bacteraemia, its incidence is increasing, especially that of *E. faecium*, which presents a worrisome annual increase of 19% in Europe [3]. Enterococcal BSI mortality ranges from 14–50%, being higher in the case of *E. faecium* compared to *E. faecalis* [2,4,5]. Specifically, ampicillin resistant and glycopeptide susceptible *E. faecium* (GSEF)-BSI mortality is 25–35% [6], whereas vancomycin resistant strains present a higher mortality [7].

Among the potential reasons for their success in the hospital remain their intrinsic resistance to many antimicrobials, their capacity to acquire new resistant traits, their extraordinary capacity to survive in hostile environments (they can survive for long periods on medical equipment, bed rails and doorknobs) and their genome plasticity [1,8]. They are in fact tolerant to disinfectants, heat, chlorine and some alcohol preparations [8]. Enterococci are resistant to many antimicrobials, including clindamycin, trimethoprim-sulfamethoxazole or aminoglycosides [1]. Whereas *E. faecalis* is normally susceptible to beta-lactams as ampicillin, *E. faecium* presents a higher antimicrobial resistance, being normally resistant to beta-lactams [1]. One of the concerns of *E. faecium* is its resistance to glycopeptides, which varies across the world [1]. In 2019, 86.8% of *E. faecium* isolates were ampicillin-resistant in Spain, whereas only 1.2% showed vancomycin resistance [9].

Despite this increasing incidence and high mortality, the treatment of choice of GSEF-BSI has not yet been elucidated. Vancomycin remains the first-line therapy based on its bactericidal action, although it presents important drawbacks: the need of intravenous infusion and therefore limitation of treatment in outpatient setting, therapeutic drug monitoring (TDM), side effects (mainly nephrotoxicity, but also red-man syndrome) and a slow bactericidal activity [10,11]. Although its bacteriostatic action, linezolid is a potential alternative although evidence on its use is scarce [6]. This oxazolidinone can be administered through oral route, although it also needs for TDM and shows an unfavourable safety profile (thrombocytopenia, anaemia, neuropathy) especially in long treatments [10]. Daptomycin is an appealing option in the treatment of bacteraemia given its rapid bactericidal action [12]. However, although daptomycin use has risen for enterococcal infections, data on the use of this antimicrobial for the treatment of GSEF-BSI are lacking [1,13,14].

Daptomycin has been mainly studied in the treatment of vancomycin-resistant *E. faecium* BSI, showing controversial results when compared to linezolid [7,13,15]. Daptomycin’s breakpoints, its optimal dose and the risk of resistance development along the treatment are some of the concerns associated with this antibiotic [14,16]. The European Committee on Antimicrobial Susceptibility Testing (EUCAST) Steering Committee recognized the remaining uncertainties of daptomycin in the treatment of enterococcal infections, advising an increased vigilance even with the use of high-dose daptomycin due to efficacy and safety concerns [14].

Therefore, the treatment with daptomycin for enterococcal infections may not be as effective as expected. Due to the concerns of potential treatment failures with this antibiotic, the objective of this study was to assess the effectiveness and safety of daptomycin in the treatment of GSEF-BSI compared to glycopeptides.

## 2. Results

A total of 192 patients presented a positive blood culture with GSEF during the study period (Table 1).

**Figure 1:**
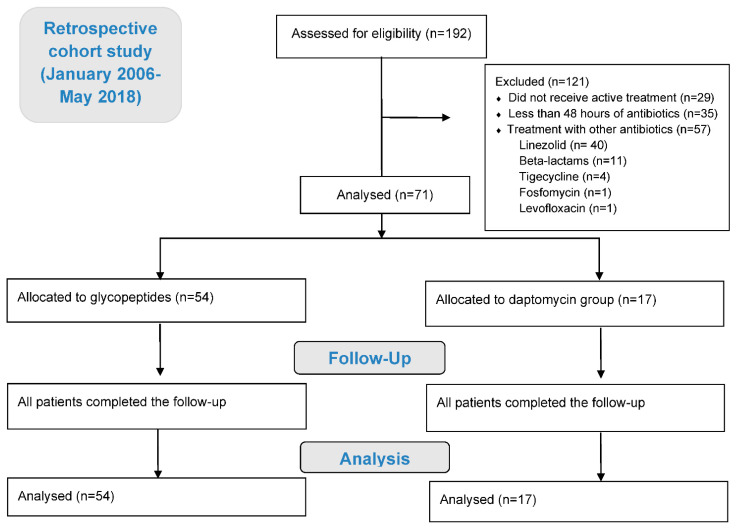
The study flow chart in line with the STROBE: 192 patients were initially assessed for eligibility, of which 121 were excluded. Finally, 71 patients were analysed, 54 in the group allocated to glycopeptides and 17 in the group allocated to daptomycin. All patients completed the follow up. STROBE: (Strengthening the Reporting of Observational Studies in Epidemiology).

One hundred and twenty-one patients were excluded as they did not receive an active treatment (29, 24.0%), received antibiotics less than 48 h (35, 28.9%), or were treated with other antibiotics (57, 47.1%). Finally, 71 (37.0%) patients were treated either with glycopeptides (54, 28.1%) or daptomycin (17, 8.9%) and were included in the final analysis. From the 54 patients treated with glycopeptides, 6 (11.1%) received teicoplanin, while the rest were treated with vancomycin. Figure 1 summarised the study flow chart.

Table 2 showed the baseline and clinical characteristics of analysed patients. The median age was 68.0 (56.0–78.0) years, most of the included patients were male and hospitalized in medical wards. Charlson Comorbidity Index was 2.0 (0–2.0), with both groups presenting similar comorbidities excepting for a higher incidence of chronic kidney disease in the daptomycin group (9.9% vs. 47.1%, *p* = 0.005).

Concerning clinical presentation, 8 (11.3%) patients were diagnosed of septic shock and 10 (14.1%) were mechanically ventilated. SOFA and Pitt bacteraemia scores were 3.0 (1.0–5.0) and 1.0 (0.0–2.0), respectively. No differences were found in the clinical presentation, apart from a worse creatinine and glomerular filtration rate in the daptomycin group. Most of the bacteraemia were of high risk, being the main sources of infection intra-abdominal, biliary and catheter related.

The incidence of polymicrobial bacteraemia was similar among groups (27.8% vs. 35.3%, *p* = 0.556). The main concomitant microorganisms were *Enterobacterales*, (66.7% vs. 50.0%, *p* = 1.000), followed by other gram-positive cocci (20.0% vs. 0%, *p* = 1.000) and *Candida* spp. (0% vs. 1%, *p* = 0.105). All these patients received adequate in vitro antimicrobial therapy to treat these microorganisms.

No differences were found in the inadequate empirical treatment rate between both cohorts (72.2% vs. 52.9%, *p* = 0.200), nor in the time until appropriate treatment: 2 (1.0–4.0) vs. 2 (0–4.0) days (*p* = 0.907).

Among the 71 isolates, 4 (5.6%) were susceptible to ampicillin but were treated with either with glycopeptides (2, 50.0%) or daptomycin (2, 50.0%). The median minimum inhibitory concentration (MIC) of daptomycin was of 1.0 (0.5–2.0) mg/L (clinical breakpoint of EUCAST: insufficient evidence [14]), whereas that of vancomycin was 1.0 (0.5–2.0) mg/L (clinical breakpoint of EUCAST: 4 mg/L [17]).

Daptomycin dosage was 500.0 (375.0–700.0) mg/day [7.8 (5.8–9.2) mg/kg/day] and was prescribed for a total of 6.0 (3.5–11.5) days. Vancomycin was administered at a dose of 2000 (1625–2000) mg/day [29.9 (19.4–34.2) mg/kg/day] for 12.0 (6.0–14.8) days. Vancomycin through levels were 10.8 (8.2–17.6) mg/L. All patients in the teicoplanin group received 6 mg/kg/12h three doses and then 6 mg/kg/24h. Teicoplanin dosage was 400.0 (400.0–650.0) mg/day (6.4 (5.0–9.7) mg/kg/day), with a treatment duration of 9.5 (8.8–18.0) days.

From a total of 5 patients diagnosed of urinary-tract GSEF-BSI, 4 cases (all in the daptomycin group) presented an indwelling catheter on the day of the episode and was removed in all of them. Similarly, line was removed in all the patients diagnosed of intravenous line-related BSI. Concerning intra-abdominal, biliary, and wound infections, there were no differences in the surgical source control among groups (*p* = 1.000).

### 2.1. Clinical Outcomes

Patients treated with daptomycin presented lower clinical cure compared to those treated with glycopeptides [10 (58.8%) vs. 45 (83.3%), relative risk (RR) 0.416 (95% CI, 0.189–0.915), *p* = 0.048]. In the multivariate analysis, after adjusting for chronic kidney disease, creatinine, and infectious foci (high versus low risk), the clinical cure was significantly higher with glycopeptides (aOR 4.313, 95% CI, 1.053–17.660). Main outcomes were summarised in Table 3.

The reason for treatment failure was the lack of clinical improvement according to the responsible physician criteria in all the cases. No differences were found in terms of time to defervescence, 14-day, 30-day, and in-hospital mortality, although 14-day mortality was lower in the glycopeptides group (1 (1.9%) vs. 2 (11.8%), RR 0.331 (95% CI 0.132–0.827)).

Patients treated with glycopeptides showed higher microbiological eradication compared to those treated with daptomycin (51 (94.4%) vs. 11 (64.7%), RR 3.758 (95% CI, 1.852–7.624), *p* = 0.005). These results were maintained in the multivariate analysis after adjusting for chronic kidney disease, creatinine, and infectious foci, with an aOR of 14.766 (95% CI 2.305–94.588). Hospital length of stay in patients treated with daptomycin was higher [39.0 (21.8–61.3) vs. 48.0 (29.0–122.5), *p* = 0.133], although did not achieve statistical significance. No other differences were found concerning relapse, superinfection, or readmissions.

### 2.2. Safety

Glycopeptides and daptomycin presented a similar rate of adverse events or need for treatment discontinuation. Patients treated with glycopeptides presented a higher incidence of gastrointestinal side effects (nausea and vomiting: 5.8% vs. 0%, *p* = 1.000; diarrhoea 7.7% vs. 0%, *p* = 0.566). The incidence of nephrotoxicity (16.0% vs. 6.3%, *p* = 0.436), thrombocytopenia (28.0% vs. 31.3%, *p* = 0.743), anaemia (8.2% vs. 0%, *p* = 0.565), and creatinine phosphokinase elevation (33.3% vs. 0%, *p* = 1.000) was similar among groups. No other rarer events as red-man syndrome, rhabdomyolysis or eosinophilic pneumonia were reported. The incidence of side effects was shown in Table 4.

## 3. Discussion

The increase in the incidence of *E. faecium* bacteraemia as well as its high mortality make the study of this disease essential [3]. In this work we tried to elucidate the existing controversies about the use of daptomycin for GSEF-BSI. To the best of our knowledge, this is the first study assessing the treatment outcomes of daptomycin in the treatment of GSEF-BSI. In our study, the treatment with daptomycin showed a lower rate of clinical cure compared to glycopeptides, mainly due to the lack of clinical improvement. However, the mortality rate was similar between both groups.

Concerns about the effectiveness of daptomycin in the treatment of enterococcal bacteraemia have already been discussed based on available data with vancomycin-resistant strains [14]. In two meta-analyses of retrospective observational studies comparing daptomycin and linezolid for the treatment of vancomycin-resistant *E. faecium* bacteraemia, linezolid seemed to present a lower 30-day mortality, but these results were challenged in the highest retrospective observational study published where this oxazolidinone was associated with a higher risk of treatment failure and 30-day mortality [7,15]. Unfortunately, data on the outcomes of different treatments of GSEF-BSI are scarce, and the outcomes of daptomycin in this setting have never been studied.

In our study, although the small sample size, a higher risk of treatment failure was found in patients treated with daptomycin. Furthermore, a higher incidence of 14-day mortality was observed, which was not statistically significant probably due to the low sample size. Several reasons may explain these findings: daptomycin dosage, the breakpoints, and the risk of resistance development [14].

The optimal dosage of daptomycin for enterococcal bacteraemia remains unknown. It is likely that even with high doses (10–12 mg/kg/day) required pharmacokinetic/pharmacodynamic (PK/PD) targets are unachievable without safety issues with MICs > 2 mg/L [14]. From a PK/PD perspective, a free drug area under the plasma concentration–time curve to MIC ratio (fAUC/MIC) > 27.43 h.mg/L has been associated with 30-day survival in low acuity patients [18,19]. A dose of 6 mg/kg/day achieved a probability target attainment (PTA) > 90% only with MICs ≤ 1 mg/L, whereas an optimal PTA for a MIC of 2 mg/L could only be achieved with 12 mg/kg/day [18]. Higher rates of microbiological failure and mortality have been reported in BSIs caused by *E. faecium* strains with daptomycin MIC 3–4 mg/L when treated with daptomycin [20,21]. In fact, two recent studies found that no dose presented an acceptable PTA against a MIC of 4 mg/L in the Monte Carlo simulation [22,23]. When MICs of 2–4 mg/L were analysed, a fixed dose of 1500 mg daily was needed for ≥ 90% PTA, but this dosage carried an undue risk of toxicity as was associated with an increased 24.5–80.5% risk of achieving a Cmin ≥ 24.3 mg/L [24].

Based on these data, the CLSI revised the daptomycin breakpoints for *Enterococcus* spp. and established that all the MICs ≤ 4 mg/L should be reported as SDD (susceptible dose dependent), based on a dosage regimens of 8–12 mg/kg/day [25]. The EUCAST changed their breakpoints to insufficient evidence [14].

In our study the median daptomycin dose was near 8 mg/kg/day. Although this dose could play a role in the outcomes as doses > 9 mg/kg/day were related to a survival benefit and the risk of emergence of resistance [16,26,27], the CLSI recommends 8–12 mg/kg/day [25]. In addition, the median MIC was 1 mg/L, so this dosage may have been sufficient.

Another important drawback of daptomycin is the resistance development during the treatment, mainly due to changes in the *LiaFSR* regulatory system [28]. Eighty percent of *E. faecium* isolates with MICs between 3–4 mg/L harbour changes in this system, whereas these substitutions are rarely found in strains with lower MICs [28]. The size of the inoculum may also play a role [27].

To overcome these limitations, the use of combination therapy with β-lactams has been recommended based on in vitro data [29]. The treatment with these family of antibiotics may reduce the MIC to daptomycin, facilitating the achievement of optimal PK/PD indexes even in daptomycin-resistant strains, through the use of lower doses with an acceptable safety risk [28,29]. The addition of ampicillin to daptomycin resulted in bactericidal activity event at lower doses of daptomycin (8–10 mg/kg) in an in vitro biofilm model [28]. A murine model found that the addition of ampicillin in continuous infusion (but no ertapenem or ceftaroline) to daptomycin was effective, synergistic and prevented the development of resistance in the treatment of multidrug-resistant *E. faecium* infections [29]. Caution is nevertheless advised since the benefits of this synergy may be strain dependent and therefore not universal [28].

In our study, patients treated with vancomycin presented a higher incidence of side effects, mainly due to gastrointestinal side effects and nephrotoxicity. However, the need for treatment discontinuation was not different. The difference in the length of treatment may have played a role in these differences.

Based on the results of our study daptomycin was associated with limitations, and until evidence from randomised controlled clinical trials are available, vancomycin should be preferred over daptomycin for the treatment of GSEF-BSI. In cases of intolerance to glycopeptides, linezolid could be considered if a deep-seated infection (endocarditis, thrombophlebitis) is ruled out. Although some concerns have been raised due to its bacteriostatic activity, linezolid has proven to be similar to vancomycin in terms of effectiveness and safety in the treatment of GSEF-BSI or *Staphylococcus aureus* bacteraemia [6]. Dalbavancin and oritavancin are other potential options although clinical evidence on their use is still scarce [30,31,32]. Daptomycin should only be used in the treatment of GSEF-BSI when no treatment options (glycopeptides, linezolid) remain available or deep-seated infections occur. In these exceptional cases, doses of up to 12 mg/kg/daily, a close toxicity and therapeutic drug monitoring, an appropriate MIC determination and combination with continuous infusion of ampicillin could be considered to try to optimize treatment outcomes.

This study presents several limitations. It consists of a non-randomised single-centre retrospective observational study, which is subject to their typical drawbacks. The indication bias could be present, although no significant differences were found in baseline characteristics excepting from renal function. We acknowledge that a propensity-score analysis would have improved the quality of our work, although the low sample size prevented us from carrying out such analysis. The existence of other potential confounding factors not registered must be also considered. As expected, a significant incidence of polymicrobial bacteraemia was observed which may have influenced the outcomes. However, due to the difficulty of finding patients with monomicrobial bacteraemia a mixed analysis was performed, with all the concomitant microorganisms appropriately treated. We also acknowledge the small sample size. However, we were limited by the incidence of this infection in our centre. The number of included patients was low compared to the number of years studied, but only 192 patients had at least one positive culture during the studied period. Of this population, 121 had to be discarded based on the exclusion criteria. Finally, throughout the entire period only 17 patients were treated with daptomycin. Although the power of the study is limited by its small sample size, the finding of statistically significant results suggests that these findings could be maintained with larger samples.

## 4. Materials and Methods

### 4.1. Study Design and Setting

This was a retrospective observational cohort study conducted from January 2006 to May 2018 at the Hospital del Mar, a 420-bed university tertiary care hospital located in Barcelona, Spain. The hospital includes two different intensive care units (ICU) with a program for renal transplantation. All patients were followed-up for 3 months by electronic records after hospital discharge or death. This study was reviewed and approved by the ethics committee of the institution and followed the Strengthening The Reporting of Observational Studies in Epidemiology (STROBE) statement guidelines [33].

### 4.2. Participants

We identified all adult patients (>18 years) with at least 1 positive blood culture for GSEF through computer-generated microbiological data. Thereafter, electronic records were reviewed, and patients were selected if they were treated more than 48 h with glycopeptides (vancomycin/teicoplanin) or daptomycin according to the responsible physician criteria. Patients were excluded if they did not receive antibiotic therapy or if they received another antibiotic against GSEF. In patients with multiple episodes only the first episode was included.

Daptomycin, vancomycin and teicoplanin dosages were adjusted by the responsible physician according to the weight and renal function, with the possible recommendation of the infectious diseases’ pharmacist or physician. Vancomycin plasmatic levels were performed throughout the study period and were adjusted by clinical pharmacists.

### 4.3. Variables and Data Sources

Epidemiological data included age, sex, body mass index, hospitalization ward (medical or surgical), site of acquisition (community-acquired, healthcare-associated or nosocomial [34]), underlying diseases and their severity according to Charlson index score [35], immunosuppression, neutropenia and presence of devices (vascular or urinary catheter, drainages). Clinical data included the source of bacteraemia, severity of illness and antimicrobial therapy. Severity of illness was assessed through the Pitt bacteraemia score [36], Sepsis-related Organ Failure Assessment [37], diagnosis of sepsis and septic shock according to the 2016 definitions [38] and need for mechanical ventilation on the day of the episode. In terms of antimicrobial therapy, empirical therapy, definitive treatment, time to adequate therapy, length of definitive treatment and antibiotic-related adverse events were recorded. All the variables were obtained by reviewing the electronic chart records.

### 4.4. Definitions

Bacteraemia was defined according to the Centre for Disease Control and Prevention guidance [39]. Immunosuppression was considered in patients receiving chemotherapy, radiotherapy or other immunosuppressive drugs including corticosteroids during the previous month. The source of bacteraemia was defined following both clinical and microbiological criteria [39,40], and was classified into two groups: low risk (urinary tract, vascular catheter and biliary tract) and high risk (all the others) [41].

Empiric therapy was deemed appropriate when at least one of the antibiotics was active in vitro against *E. faecium* [41]. Time to adequate therapy was defined as the days until receiving an appropriate treatment.

Clinical cure was considered when patients presented a resolution of signs and symptoms of infection [13]. For this purpose, we also evaluated laboratory parameters as C-reactive protein, procalcitonin, leucocytes and neutrophils. Otherwise, treatment failure was defined as the need for treatment change due to the persistence of infection, death or toxicity [13]. Microbiological eradication was recorded when the last blood culture drawn after initiation of therapy was negative [13] or, if not available, when clinical cure/improvement were achieved [6]. Persistent bacteraemia was defined as the isolation of GSEF in blood cultures after > 72 h of adequate treatment and relapse was defined as the isolation of positive blood culture after documented clearance within 30 days of index culture [6]. Readmissions were recorded up to 3 months after the discharge. Side events were defined as the development of an adverse event proven or suspected to be related to the agent used after the initiation of the therapy [13]. Side effects were recorded according to the information available in the medical records or analytics. Renal toxicity was assessed through the RIFLE criteria [42], whereas thrombocytopenia was defined as the decrease in the platelet count to < 75% from the baseline [6]. Anaemia was considered when a reduction ≥ 2 g/dL in haemoglobin concentration was developed [6]. Creatine phosphokinases (CPT) were measured at the discretion of the responsible physician. CPK elevation was considered as an elevated value ≥ 3 times the upper limit of normal in those with normal baseline CPK and ≥ 5 times the in those with elevated baseline CPK [24].

The primary outcome was the assessment of clinical cure at the end of the treatment. Secondary outcomes included 14-day, 30-day and in-hospital mortality, time to defervescence (time until fever returns to normal temperature), microbiological eradication, relapse, and superinfection. Finally, hospital length of stay, readmission rates and treatment-related side effects were also recorded.

### 4.5. Microbiology

Polymicrobial bacteraemia was considered when at least 1 non *E. faecium* species were isolated from the same blood culture than the enterococci [43]. The appropriateness of antibiotic therapy against these concomitant microorganisms was recorded, considering adequate when was active in vitro.

The EUCAST clinical breakpoints were applicated to determine the susceptibility to the different antimicrobials [17]. The full susceptibility test was performed by microdilution (MicroScan©, Mahwah, NJ, USA). The MIC of vancomycin, teicoplanin and daptomycin were confirmed by gradient diffusion (E-test, bioMérieux©, Marcy-l’Etoile, France).

### 4.6. Statistical Analysis

The sample size was limited by the low number of patients that received daptomycin over the study period. After the normality test of Kolmogorov–Smirnov, all the statistical analyses were performed with non-parametrical tests. In the case of categorical variables, these were presented as absolute numbers (percentages) and were compared using Fisher exact tests. Continuous variables were described as median (Q1–Q3) and were analysed by Mann–Whitney U test. Multivariate analysis was performed using a forward stepwise logistic regression model.

A *p* < 0.05 was considered statistically significant in all the analyses. Statistical analyses were performed with IBM SPSS Statistics 22.0 (IBM Co., Armonk, NY, USA).

## 5. Conclusions

Treatment with daptomycin compared to glycopeptides resulted in a lower clinical cure for GSEF-BSI, results that persisted after adjusting for confounding factors in the multivariate analysis. Treatment with this lipopeptide was also associated with a lower microbiological eradication. Both treatments showed a similar safety profile. Randomised controlled trials are needed to determine the role of daptomycin in the treatment of GSEF-BSI.

## Figures and Tables

**Table 1:** Baseline and clinical characteristics of the 192 patients diagnosed of *Enterococcus faecium* bacteraemia.

	Whole Cohort (*n* = 192)
Age, years	69.0 (64.0–78.0)
<50 years	19 (9.9)
50–75 years	105 (54.7)
>75 years	68 (35.4)
Female	54 (28.1)
Weight, kg	68.6 (59.0–80.2)
Body Mass Index, kg/m^2^	24.6 (23.0–29.1)
*Medical/surgical status*	
Medical status	114 (59.4)
Surgical status	78 (40.6)
*Means of acquisition*	
Community acquired	17 (8.9)
Healthcare acquired	35 (18.2)
Nosocomial	140 (72.9)
*Comorbidities*	
Charlson comorbidity index	2.0 (0.0–2.0)
Diabetes Mellitus	77 (39.6)
Arterial hypertension	114 (59.4)
Cardiopathy	53 (27.6)
Liver cirrhosis	19 (9.9)
Solid tumor	58 (30.2)
Renal transplantation	11 (5.7)
Immunosuppression	86 (44.8)
Chemotherapy	33 (17.2)
Corticosteroids	72 (37.5)
Chronic kidney disease	36 (18.8)
*Clinical presentation*	
Septic shock	56 (29.2)
SOFA score	3.0 (1.0–6.0)
SOFA > 2	124 (64.5)
Vasoactive drugs	52 (27.1)
Mechanical ventilation	47 (24.5)
Pitt bacteraemia score	2.0 (1.0–3.0)
Creatinine, mg/dL	0.9 (0.6–1.4)
Glomerular filtration rate, mL/min/1.73 m^2^	79.1 (46.0–99.3)
Albumin, g/dL	2.7 (2.2–3.2)
*Source of bacteraemia*	
*High risk*	*110 (57.3)*
Abdominal	62 (32.3)
Unknown	25 (13.0)
Respiratory tract	10 (5.2)
Endocarditis	1 (0.5)
Thrombophlebitis	4 (2.1)
Skin and soft tissue	8 (4.2)
*Low risk*	*82 (42.7)*
Urinary tract	18 (9.4)
Catheter related	26 (13.5)
Biliary	38 (19.8)

Data are represented as median (Q1–Q3) or in absolute numbers (percentage).

**Table 2:** Baseline and clinical characteristics of patients diagnosed of *Enterococcus faecium* bacteraemia treated with glycopeptides or daptomycin.

	Glycopeptides (*n* = 54)	Daptomycin (*n* = 17)	*p* Value
Age, years	67.5 (55.0–74.5)	74.0 (62.5–84.5)	0.166
<50 years	8 (14.8)	2 (11.8)	
50–75 years	33 (61.1)	7 (41.2)	
>75 years	13 (24.1)	8 (47.1)	
Female	17 (31.5)	7 (41.2)	0.559
Weight, kg	65.0 (58.0–80.0)	70.0 (61.0–80.0)	0.350
Body Mass Index, kg/m^2^	24.6 (21.0–29.0)	25.2 (21.1–31.2)	0.450
*Medical/surgical status*			0.572
Medical status	36 (66.7)	10 (58.8)	
Surgical status	18 (33.3)	7 (41.2)	
*Means of acquisition*			1.000
Community acquired	2 (3.7)	0	
Healthcare acquired	12 (22.2)	4 (23.5)	
Nosocomial	40 (74.1)	13 (76.5)	
*Comorbidities*			
Charlson comorbidity index	2.0 (0.3–2.0)	1.0 (0–2.0)	0.222
Diabetes Mellitus	18 (33.3)	9 (52.9)	0.164
Arterial hypertension	29 (53.7)	11 (64.7)	0.577
Cardiopathy	12 (22.2)	7 (41.2)	0.207
Liver cirrhosis	7 (13.0)	2 (11.8)	1.000
Solid tumor	20 (37.0)	6 (35.3)	1.000
Renal transplantation	2 (3.7)	2 (11.8)	0.241
Immunosuppression	25 (46.3)	8 (47.1)	1.000
Chemotherapy	11 (20.4)	4 (23.5)	0.745
Corticosteroids	22 (40.7)	5 (29.4)	0.745
Chronic kidney disease	11 (9.9)	8 (47.1)	0.005
*Clinical presentation*			
Septic shock	7 (13.0)	1 (5.9)	0.670
SOFA score	3.0 (1.0–5.0)	2.0 (1.0–6.5)	0.724
SOFA > 2	28 (51.9)	9 (52.9)	1.000
Vasoactive drugs	7 (13.0)	3 (17.6)	0.694
Mechanical ventilation	6 (11.1)	4 (23.5)	0.237
Pitt bacteraemia score	1.0 (0–2.0)	2.0 (0.5–3.5)	0.181
Creatinine, mg/dL	0.8 (0.6–1.0)	1.2 (0.7–2.1)	0.009
Glomerular filtration rate, mL/min/1.73 m^2^	86.3 (66.6–104.4)	59.0 (25.3–90.6)	0.018
Albumin, g/dL	2.7 (2.3–3.3)	2.6 (2.1–3.4)	0.842
*Source of bacteraemia*			
*High risk*	*33 (61.1)*	*7 (41.2)*	*0.160*
Abdominal	22 (40.7)	1 (5.9)	
Unknown	5 (9.3)	4 (23.5)	
Respiratory tract	1 (1.9)	0 (0)	
Endocarditis	1 (1.9)	0 (0)	
Thrombophlebitis	2 (3.7)	1 (5.9)	
Skin and soft tissue	2 (3.7)	1 (5.9)	
*Low risk*	*21 (38.9)*	*10 (58.8)*	
Urinary tract	1 (1.7)	4 (23.5)	
Catheter related	7 (13.0)	3 (17.6)	
Biliary	11 (20.4)	3 (17.6)	

Data are represented as median (Q1–Q3) or in absolute numbers (percentage).

**Table 3:** Comparison of clinical outcomes according to treatment group in patients diagnosed of vancomycin-susceptible *Enterococcus faecium* bacteraemia.

	Glycopeptides (*n* = 54)	Daptomycin (*n* = 17)	Relative Risk (95% CI)	*p* Value
*Clinical outcomes*	**45 (83.3)**	**10 (58.8)**	0.416 (0.189–0.915)	**0.048**
Clinical cure at the end of therapy				
Time to defervescence, days	1 (0–2.0)	1 (0–2.0)	-	0.881
*Mortality*				
14-day mortality	1 (1.9)	2 (11.8)	0.331 (0.132–0.827)	0.140
30-day mortality	7 (13.2)	4 (23.5)	0.606 (0.242–1.516)	0.443
In-hospital mortality	12 (22.6)	6 (35.3)	0.645 (0.275–1.467)	0.346
*Microbiological data*				
Eradication	51 (94.4)	11 (64.7)	3.758 (1.852–7.624)	0.005
Relapse	3 (5.6)	2 (11.8)	0.568 (0.178–1.816)	0.587
Superinfection	27 (50.0)	11 (64.7)	0.628 (0.261–1.512)	0.404
Hospital LOS, days	39.0 (21.8–61.3)	48.0 (29.0–112.5)	-	0.133
Readmissions	18 (34.0)	6 (35.6)	0.957 (0.403–2.269)	1.000

Data are represented as median (Q1–Q3) or in absolute numbers (percentage). CI: confidence interval; LOS: length of stay.

**Table 4:** Side effects by antimicrobial treatment for vancomycin-susceptible *Enterococcus faecium* bacteraemia.

	Glycopeptides (*n* = 54)	Daptomycin (*n* = 17)	Relative Risk (95% CI)	*p* Value
Any side effect	26/52 (50.0)	5/16 (31.3)	1.843 (0.718–4.734)	0.254
Discontinuation of treatment	4/52 (7.7)	0 (0)	-	0.566
Nausea and vomiting	3/52 (5.8)	0 (0)	-	1.000
Diarrhoea	4/52 (7.7)	0 (0)	-	0.566
*Nephrotoxicity*	8/50 (16.0)	1/16 (6.3)	2.368 (0.355–15.807)	0.436
R	6/50 (12.0)	0 (0)		
F	1/50 (2.0)	0 (0)		
L	1/50 (2.0)	1 (5.9)		
Thrombocytopenia	14/50 (28.0)	5/16 (31.3)	0.889 (0.357–2.216)	0.743
Anaemia	4/49 (8.2)	0 (0)	-	0.565
Creatinine phosphokinase elevation	1/3 (33.3)	0/3 (0)	-	1.000

Data are represented in absolute numbers (percentage). CI: confidence interval; R: risk; F: failure; L: loss.

## Data Availability

The data presented in this study are available on request from the corresponding author. The data are not publicly available due to privacy and ethical reasons.
